# Supplementary material for: An open-source framework for large-scale, flexible evaluation of biomedical text mining systems
Source: J Biomed Discov Collab. 2008 Jan 29;3:1. doi: 10.1186/1747-5333-3-1 (PMC2276192; doi:10.1186/1747-5333-3-1)
Supplement: Additional file 1 — Evaluation platform source code. The additional file contains the software evaluation framework discussed in this paper. The most current release can be downloaded from SourceForge.net [1]. See the accompanying README file for instructions on installation and use. Also included in the distribution is a collection of UIMA wrappers for some commonly used BioNLP tools and annotated corpora. [file 1747-5333-3-1-S1.zip › bionlp_uima_repository-1.0.1/docs/api/src-html/edu/uchsc/ccp/uima/ae/util/AnnotationFromFileLoader_AE.html]

```
001    /*
002     *
003     * The Original Code is UCHSC UIMA.
004     *
005     * The Initial Developer of the Original Code is University of Colorado.  
006     * Copyright (C) 2005.  All Rights Reserved.
007     *
008     * UCHSC Utilities was developed by the Center for Computational Pharmacology
009     * (http://compbio.uchsc.edu) at the University of Colorado Health 
010     *  Sciences Center School of Medicine with support from the National 
011     *  Library of Medicine.  
012     *
013     *
014     * Contributor(s):
015     *   William A. Baumgartner <william.baumgartner@uchsc.edu> (Original Author)
016     *   
017     * Created: August 2005
018     * Dependencies:
019     * ToDo: 
020     *      This class should probably use the findSpans method in GenericTextUtils
021     * Changes:
022     *      03/10/06 - wab - Puntation (periods) are now replaced by " ." (notice the space) in the input text before searching for gene names. 
023     *                       This is in response to Jim's request because ABNER was not able to find "NF-kappa B." at 
024     *                       the end of a sentence.
025     *      02/15/06 - rjf - Added annotation back-pointer into mentions created by
026     *                        ABNER annotator
027     *      01/09/06 - wab - Made necessary changes to conform with revised Type System
028     *                     - Specifically, the new ClassMention structure
029     *      
030     */
031    package edu.uchsc.ccp.uima.ae.util;
032    
033    import java.io.BufferedReader;
034    import java.io.IOException;
035    import java.util.ArrayList;
036    import java.util.HashMap;
037    import java.util.Iterator;
038    import java.util.List;
039    import java.util.Map;
040    
041    import org.apache.uima.analysis_engine.ResultSpecification;
042    import org.apache.uima.analysis_engine.annotator.AnnotatorConfigurationException;
043    import org.apache.uima.analysis_engine.annotator.AnnotatorContext;
044    import org.apache.uima.analysis_engine.annotator.AnnotatorContextException;
045    import org.apache.uima.analysis_engine.annotator.AnnotatorInitializationException;
046    import org.apache.uima.analysis_engine.annotator.AnnotatorProcessException;
047    import org.apache.uima.analysis_engine.annotator.JTextAnnotator_ImplBase;
048    import org.apache.uima.jcas.JCas;
049    
050    import edu.uchsc.ccp.uima.annotation.CCPDocumentInformation;
051    import edu.uchsc.ccp.uima.util.UIMA_Util;
052    import edu.uchsc.ccp.util.nlp.GenericTextUtils;
053    import edu.uchsc.ccp.util.nlp.annotation.AnnotationSet;
054    import edu.uchsc.ccp.util.nlp.annotation.Annotator;
055    import edu.uchsc.ccp.util.nlp.annotation.TextAnnotation;
056    import edu.uchsc.ccp.util.nlp.mention.ClassMention;
057    
058    /**
059     * This AE reads in annotations from a file. The file may have been created by the AnnotationToFileOutputPrinter selection on the AnnotationPrinter
060     * AE. The input format for the file is one annotation per line: <br>
061     * <br>
062     * documentID|annotatorID|spanStart spanEnd|classMentionName|coveredText
063     * 
064     * @author William A. Baumgartner, Jr.
065     * 
066     */
067    public class AnnotationFromFileLoader_AE extends JTextAnnotator_ImplBase {
068        private final boolean DEBUG = false;
069    
070        public final String PARAM_ANNOTATION_FILE = "AnnotationFile";
071    
072        private Map<String, List<TextAnnotation>> documentID2AnnotationsMap;
073    
074        public void initialize(AnnotatorContext ac) throws AnnotatorInitializationException, AnnotatorConfigurationException {
075    
076            /* get the filename for the pretagged mutations file (.pprj) from the descriptor file */
077            String annotationsFile;
078            try {
079                annotationsFile = (String) ac.getConfigParameterValue(PARAM_ANNOTATION_FILE);
080    
081                /* load documentid to annotation map */
082                try {
083                    
084                    loadAnnotations(annotationsFile);
085                    System.err.println("Initializing AnnotationFromFileLoader: Loading annotations from: " + annotationsFile);
086                } catch (IOException e) {
087                    e.printStackTrace();
088                }
089            } catch (AnnotatorContextException e1) {
090                e1.printStackTrace();
091            }
092    
093            super.initialize(ac);
094        }
095    
096        public void process(JCas jcas, ResultSpecification arg1) throws AnnotatorProcessException {
097            String documentID = null;
098            Iterator it = jcas.getJFSIndexRepository().getAnnotationIndex(CCPDocumentInformation.type).iterator();
099            if (it.hasNext()) { /* there will be at most one CCPDocumentInformation annotation */
100                CCPDocumentInformation docInfo = (CCPDocumentInformation) it.next();
101                documentID = docInfo.getDocumentID();
102            }
103    
104            if (documentID2AnnotationsMap.containsKey(documentID)) {
105                List<TextAnnotation> taList = documentID2AnnotationsMap.get(documentID);
106                UIMA_Util uimaUtil = new UIMA_Util();
107                uimaUtil.putTextAnnotationsIntoJCas(jcas, taList);
108            } else {
109    //            warn("DocumentID not detected in annotationfile: " + documentID);
110            }
111        }
112    
113        private void loadAnnotations(String annotationFile) throws IOException {
114    
115            documentID2AnnotationsMap = new HashMap<String, List<TextAnnotation>>();
116    
117            BufferedReader br = GenericTextUtils.openReader(annotationFile);
118            String line;
119            int count = 0;
120            while ((line = br.readLine()) != null) {
121                // System.out.println("Loading annotation: " + count++);
122                /* parse the line */
123                String[] toks = line.split("\\|");
124                if (toks.length == 5) {
125                    String documentID = toks[0];
126                    int annotatorID = Integer.parseInt(toks[1]);
127                    String annotationType = toks[3];
128                    
129                    String[] spanToks = toks[2].split(" ");
130                    int spanStart = Integer.parseInt(spanToks[0]);
131                    int spanEnd = Integer.parseInt(spanToks[1]);
132                    String coveredText = toks[4];
133    
134                    /* create a new text annotation */
135                    TextAnnotation ta = new TextAnnotation();
136                    ta.setDocumentID(documentID);
137                    ta.setAnnotationSpanEnd(spanEnd);
138                    ta.setAnnotationSpanStart(spanStart);
139                    ta.setCoveredText(coveredText);
140    
141                    ClassMention cm = new ClassMention(annotationType);
142                    ta.setClassMention(cm);
143    
144                    
145                    Annotator annotator = new Annotator(annotatorID, "","","");
146                    ta.setAnnotator(annotator);
147    
148                    AnnotationSet tntAnnotationSet = new AnnotationSet(new Integer(-1), "Default", "Default");
149                    ta.addAnnotationSet(tntAnnotationSet);
150    
151                    /*
152                     * if the documentID is already in the Map, then add this textAnnotation to the List that is already stored, otherwise create a new
153                     * list and add it to the Map
154                     */
155    
156                    if (documentID2AnnotationsMap.containsKey(documentID)) {
157                        documentID2AnnotationsMap.get(documentID).add(ta);
158                    } else {
159                        List<TextAnnotation> taList = new ArrayList<TextAnnotation>();
160                        taList.add(ta);
161                        documentID2AnnotationsMap.put(documentID, taList);
162                    }
163    
164                } else {
165                    error(": Expected 5 items on line in annotation file, but there were: " + toks.length + "  LINE=" + line);
166                }
167    
168            }
169    
170        }
171    
172        private void error(String message) {
173            System.err.println("ERROR -- AnnotationsFromFileLoader: " + message);
174        }
175    
176        private void warn(String message) {
177            System.err.println("WARNING -- AnnotationsFromFileLoader: " + message);
178        }
179    
180        private void debug(String message) {
181            if (DEBUG) {
182                System.err.println("DEBUG -- AnnotationsFromFileLoader: " + message);
183            }
184        }
185    }
```
